# Telemedicine in Peru as a Result of the COVID-19 Pandemic: Perspective from a Country with Limited Internet Access

**DOI:** 10.4269/ajtmh.21-0255

**Published:** 2021-05-17

**Authors:** Aldo Alvarez-Risco, Shyla Del-Aguila-Arcentales, Jaime A. Yáñez

**Affiliations:** 1 Universidad de Lima, Facultad de Ciencias Empresariales y Económicas, Carrera de Negocios Internacionales, Lima, Perú;; 2 Escuela Nacional de Marina Mercante “Almirante Miguel Grau,” Callao, Peru;; 3 Universidad Peruana de Ciencias Aplicadas, Facultad de Educación, Carrera de Educación y Gestión del Aprendizaje;; 4 Teoma Global, Gerencia Corporativa de Asuntos Científicos y Regulatorios, Lima, Peru

## Abstract

The COVID-19 pandemic contributed to the worldwide implementation of telemedicine because of the need for medical care for patients, especially those with chronic diseases. This perspective paper presents the current situation of telemedicine in Peru, showing advances in regulation, cases of successful implementation, and the current challenges. Access to health should be available to all, and more efforts need to be implemented to offer access to the internet to achieve high-quality telemedicine to all the vulnerable groups in Peru.

## INTRODUCTION

The COVID-19 pandemic has reached 113 million confirmed cases and over 2.5 million deaths worldwide as of February 27, 2021.^1^ Social isolation was the main preventive measure implemented worldwide to avoid contagion,^2,3^ which caused multiple lifestyle changes. Increased heavy episodic drinking,^4^ binge eating,^5^ and development of eating disorders^6^ accompanied with a decrease in physical activity^7^ have resulted in higher body weight.^8–11^ Multiple people have lost their jobs and have experienced the death of family and friends,^12^ which has resulted in anxiety and mental distress.^13–16^ Widespread disinformation,^17^ fake news,^18^ and the anti-vaccine movement^19,20^ have caused an increase in self-medication^21^ and a general state of misinformation that has been urged to be addressed by governmental institutions.^22,23^

Telemedicine has become the preferred eHealth tool to triage physical and mental health problems without the need to physically go to a healthcare institution. Telemedicine refers to health services and information delivered or enhanced by the use of internet-related technologies.^24,25^ When COVID-19 cases started to increase worldwide there was a high number of people going to the hospitals to get tested, which increased their risk of contagion.^26,27^ As a preventive measure, dedicated phone lines were used to carry out initial symptom screening to determine if testing was necessary.^28,29^ However, because of the great demand for this service, the phone lines rapidly collapsed. Then online consultation systems were rapidly implemented as a safe online triage for suspected COVID-19 patients.^30,31^

With telemedicine, healthcare professionals have been able to transform the crisis into a safer and interactive healthcare service that has allowed them to reduce transportation time and cost due to less displacement of professionals and patients.^32,33^ Telemedicine has been used for patients with chronic diseases such as diabetes, hypertension, HIV, chronic pain,^34^ and primary immunodeficiencies.^35^ Even though telemedicine has been recognized to be of global importance,^36^ it is still new. Therefore, the healthcare professional, the patient, and the technology need to be prepared to provide a successful and high-quality service.

### Requirements for high-quality telemedicine.

To provide high-quality telemedicine services there are certain requirements that we consider need to be followed (Table 1). First, it is important to empower healthcare professionals to develop sufficient digital communication skills, which starts by acknowledging that the patient might perceive telemedicine as a lower quality of clinical care.^37^ It also needs to be acknowledged that telemedicine tends to be more accepted by younger patients who are more experienced and familiar with technology and online communication.^38^ Healthcare professionals need to acknowledge the innate limitations of telemedicine, such as barriers in reading body language, lack of physical contact,^39,40^ low confidence in the use of technology by patient and healthcare professional,^41^ technical challenges,^41^ and greater clinical re-attendance.^42^ These limitations could be minimized by providing information to the patients ahead of time, for instance providing the software or online platform to use so that the patient can become familiar with it before the consultation. It is also important to mention the likely duration of the consultation and guide them to an appropriate physical location because they will release private information or might need to disrobe as part of the examination.^24,39^ All these considerations are important because the patients might be feeling anxious about their health problems and because they are unfamiliar with the technology.

**Table 1:** Requirements to ensure the quality of telemedicine programs

Requirement 1: Empower healthcare professionals
Develop sufficient digital communication skills
Acknowledge and circumvent the innate limitations of telemedicine
Provide information to patient ahead of time
Requirement 2: Empower patients
Increase their digital health literacy
Adequate preparedness ahead of time

Second, patients need to be empowered by increasing their digital health literacy, which consists in the ability to search, find, understand, and evaluate health information from electronic sources and apply knowledge acquired to address or solve a health problem.^43^ The patient needs to have sufficient technological skills to communicate efficiently with healthcare professionals through the various available technological platforms for making health decisions.^44^ Another important requirement is that patients prepare ahead of time because they will need to provide their symptoms, medical history, questions, and concerns.^45^ As mentioned above, it is critical that the patient becomes familiar with the software or online platform to be used and chooses a proper physical location during the consultation.

Recently, a masterclass was published that exemplifies the actual wording to use for a telemedicine consultation considering the critical components that need to be present, such as greeting, introduction, courtesy, equipment check, establish remote experience, consent, signpost, preparation check, ID check, reason for call, agenda-setting, and key clinical question.^24^

### Telemedicine in chronic diseases.

Because of the pandemic, patients with chronic pain have had to deal alone with their pain and the physical limitations generated by it, which has triggered self-medication^46^ without proper medical guidance to evaluate and monitor their symptoms.^47^ However, telemedicine has demonstrated useful in chronic pain patients where significant effects on disability/interference outcomes and pain intensity were observed.^48^ Also, Furlan et al.^49^ showed a high rate of satisfaction based on healthcare professionals’ feedback related to the effective management of chronic pain using videoconferencing. Likewise, Mariano et al.^50^ described the benefits of remote groups for pain management that resulted in pain and mood improvement and in lowering patients’ feeling of disability.

Diabetes patients are a high-risk group that have experienced limitations in their routine to monitor their A1C and fasting blood glucose values. Telemedicine services have had a great impact on these patients during the COVID-19 pandemic.^51,52^ Telemedicine has allowed physicians to monitor their patients closely and to remind the patients to go to the laboratory to check their A1C values. Due to this close and constant monitoring, it has been reported that telemedicine helped in avoiding diabetic ketoacidosis admissions during the COVID-19 pandemic,^53^ which led to the development of a physicians’ guide for the provision of telemedicine in patients with diabetes.^54^

In patients with cardiovascular diseases, the advantages of telemedicine have become evident^55^ because the barriers to get continuous medical appointments were overcome by telemedicine, generating an increase in the adherence of patients with arterial hypertension and decreasing the incidence of heart failure.^56^ This is critical because patients with cardiovascular diseases require compliance with their pharmacological treatment.^57–59^ Likewise, the advantageous use of telemedicine has been reported in other conditions, such as kidney transplant.^60^ It was observed that telemedicine helped diagnose, triage, and manage transplant patients who tested positive for COVID-19.^60^

For osteoporosis patients, remote consultations have been described as particularly appropriate for disease reviews and consultations where physical examination is not required.^61^ Likewise, telemedicine has been found important in patients with pneumonia to avoid spreading the disease to health personnel and other patients.^62^ Telemedicine services have been reported to lead to faster stroke diagnoses, more frequent administration of tissue plasminogen activator, and improved long-term results.^63^

Despite the great benefits from the use of telemedicine, currently there are barriers, such as regulation, technology, a lack of trained healthcare professionals, and patients’ access to the internet.^64^ Furthermore, various factors have been identified to affect the effective and efficient use of telemedicine technologies,^65,66^ including 1) the quality of the equipment (hardware, software, and internet connection) to be used for teleconsultation; 2) the location where the consultation would take place regarding sound quality, the presence of interruptions, and the quality and reliability of internet connection; 3) the lack of experienced users to help especially elder patients, which correlates with 4) experience in using technology.^65,66^ This can also be aggravated by poverty, poor engagement, and barriers to digital health literacy,^67–69^ which are factors that complicate telemedicine in countries such as Peru.

### Telemedicine in Peru.

The health system in Peru is divided in Social Security Hospitals (SSHs), Federal Government Hospitals (FGHs), and private hospitals. In 2005 Peru approved the National Telehealth Plan by Supreme Decree No. 028-2005-MTC, which constituted the first regulatory document related to telemedicine.^70^ Four years later, in 2009, the Telehealth Technical Health Standard^71^ was approved, aimed at setting the guidelines for these services. The document details all the telemedicine options that can be provided, such as teleprevention, telediagnosis, telemonitoring, teleconsultation, telemergencies, and epidemiological televigilance in cardiology, surgery, dermatology, imaging, ophthalmology, oncology, pathology, and psychiatry. However, it was not until 2016 that the Law No. 30421 was approved to set the framework for telemedicine services.^72^ The e-prescriptions, which can solve usual prescriptions errors reported previously,^73^ were approved by legislative decree No. 1490,^74^ and it is still unknown how the physicians and pharmacists are adapting to e-prescriptions and electronic medical records in Peru.

The first reported case of COVID-19 in Peru was on March 6, 2020, with a total lockdown announced on March 16, 2020.^75^ On March 30, Peru rapidly implemented teleconsultation and telemonitoring services, establishing the necessary criteria for these services.^76^ Then on May 10, 2020, the telemedicine framework was revised to allow digital drug prescriptions and to allow the online transfer of personal data and medical history between healthcare institutions, insurance companies, and pharmacies.^74^ This set an important milestone in the right direction for an integrated national telemedicine system. However, Peru was forced to rapidly implement a telemedicine system without a proper and sufficient internet system nationwide.^77^ Because of the Andes Mountains, Peru has three geographic regions: the coast (Costa), the Peruvian Andes (Sierra), and the Jungle (Selva).^78^ This geographical division is accompanied by marked differences in access, roads, urbanization, population distribution, and health and education services. This results in marked differences in health conditions, with the coast, Andes, and rainforest having under-five mortality rates of 26%, 39%, and 42%, respectively.^78^ This is in part due to different behaviors toward urgency for medical treatment, the prevalence of pharmacological use, and self-medication practices.^21^ It comes as no surprise that there are marked differences in internet access, with 63.3% of the population in the coast having access to internet, 36% in the Peruvian Andes, and 33% in the jungle.^77^ Regardless of the limited connectivity, Peru is the country with the highest price for internet use in Latin America.^79,80^ All these factors pose a tremendous technological barrier for a nationwide telemedicine service.

Another reported barrier is that almost 60% of the population in Peru belong to the lowest socioeconomic strata, which prevents them from owning a computer at home or a smartphone with internet connection.^81^ This results in a large part of the population lacking the necessary basic technology skills for a teleconsultation. It has been proposed that, in addition to advancing the regulations related to telemedicine, we need to make technology available to the lower socioeconomic strata of Peru.^82^

On March 17, 2020, one day after the announcement of lockdown in Peru, it was announced that patients with diseases such as cancer, diabetes, tuberculosis, and HIV/AIDS were to receive preferential attention in SSHs and FGHs 24 hours a day.^83^ Furthermore, it was announced that their medications could be delivered directly to their home, with coordination with their attending physician, to reduce the risk of contagion due to their compromised immune system.^83^ On the other hand, various efforts to facilitate patient monitoring during the COVID-19 pandemic were developed. In April 2020, the Ministry of Health of Peru launched the telemedicine website “Teleatiendo” to request online consultations, which received over 4,300 requests on the first 2 weeks post-launch.^84^ The Peruvian government created the app “Peru in your hands,” which allowed medical personnel to know the areas where there was a greater probability of contagion by COVID-19 using location services.^85^ The “CoronaIsh” app was released in different regions in Peru to allow medical personnel to monitor suspected cases of COVID-19.^86^ Similarly, the Peruvian startup Smart Doctor announced its partnership with the Peruvian Ministry of Health to monitor COVID-19 patients during quarantine.^87^ The remote management of oncological patients was first assessed on 2018 in a pilot study with the mobile app ONCOpeds, which reported that use of the app improved diagnosis and referral time in pediatric cancer patients.^88^ However, this app remained as a pilot and was never launch to the general public. Another important application of telemedicine in Peru has been the monitoring of tuberculosis cases.^89,90^ Peru modified the Directorate for Tuberculosis Prevention and Control by recommending to monitor Direct Observed Therapy using telemedicine during the COVID-19 pandemic to ensure adherence to treatment and to prevent transmission to personnel.^91^

During the first months of the pandemic, there was a delay in the diagnosis and treatment of thousands of patients with pathologies like cancer because the management of COVID-19 patients was priority.^92^ The oncology areas in private clinics in Peru were gradually and partially opened to treat cancer patients while preventing them from getting infected with SARS-CoV-2.^92^ To fulfill this objective, a guidance was launched for the management of cancer patients^93^ detailing the biosecurity protocols, the protocol for online and phone consultations, and the considerations to have for chemotherapy treatment and surgical procedures.^92^

Similar efforts have been implemented for diabetic patients to continue monitoring their disease during the pandemic.^94^ The Cayetano Heredia National Hospital implemented a dedicated teleconsultation system for diabetic patients^95^ based on the published directive for the teleconsultation, telemonitoring, and teleorientation of chronic patients published by the Peruvian public health system EsSalud.^96^ The implementation of this dedicated teleconsultation has resulted in the 14% reduction of complications with an acceptable satisfaction and user understanding.^97^ However, it still needs to be evaluated in Peru if the frequency of use of telemedicine, patient and physician satisfaction, and understanding vary between the SSHs, FGHs, and private hospitals, which typically carry patients from different socioeconomic strata.

All these efforts have tremendously improved the remote care and monitoring of chronic patients in Peru; however, there is a need for a specific regulatory framework for mobile health (mHealth) apps in Peru. This was recognized in 2019 after an evaluation of various apps identifying issues related to security and privacy, quality of information provided, and lack of available evidence of their usability and effectiveness, as well as the lack of a national repository for these apps.^98^ Another important concern is that the majority of telemedicine reports are from urban areas of major cities in Peru. As mentioned above, the majority of Peruvians do not have access to internet and telemedicine; thus, internet access to rural areas in Peru should be prioritized. Internet access in rural areas has been achieved previously using WiLD multihop network, providing 3G services to eight villages on the margin of the Napo river in the rural Peruvian Amazon.^99^ This could allow Peru to implement telemedicine programs similar to the ones in Brazil and Colombia to combat malnutrition among pregnant women, mothers, and babies in the rural Amazonian forest.^100^

## CONCLUSIONS

The COVID-19 pandemic created an opportunity to expand telemedicine services, but it is necessary to evaluate in detail both the type of service that is implemented and the minimum conditions that patients need to be able to access said health services. We foresee that telemedicine in Peru could help fortify disease prevention programs, monitor chronic disease patients, and combat malnutrition in vulnerable populations, such as a pregnant women and children under 5 years old. Peru was forced into telemedicine due to the pandemic, and the government is working to improve Internet coverage. Peru is about to elect a new President and Congress, and the majority of the candidates have promised to make internet accessible to all because they have acknowledged that, regardless of the pandemic, internet connectivity can improve access to health services to vulnerable groups and the general public in Peru.
